# Description of the molecular and clinical characteristics of the mucopolysaccharidosis type VII Iberian cohort

**DOI:** 10.1186/s13023-021-02063-1

**Published:** 2021-10-22

**Authors:** Antonio Gónzalez-Meneses, Mercè Pineda, Anabela Bandeira, Patrícia Janeiro, María Ángeles Ruiz, Luisa Diogo, Ramón Cancho-Candela

**Affiliations:** 1 Unidad de Dismorfología Y Metabolismo, Hospital Universitario Virgen del Rocío, Avda. Manuel Siurot, s/n, 41013 Seville, Spain; 2 Fundació Hospital Sant Joan de Deu, Esplugues/Clínica Teknon, Barcelona, Spain; 3 Centro de Referência de Doenças Hereditárias Do Metabolismo, CHUP, Porto, Portugal; 4 Centro de Referência de Doenças Hereditárias Do Metabolismo, CHULN, Lisboa, Portugal; 5 Neuropediatría. Hospital Son Espases, Palma de Mallorca, Spain; 6 Centro de Referência de Doenças Hereditárias do Metabolismo, CHUC, Coimbra, Portugal; 7 Unidad Neurología Pediátrica, Servicio de Pediatría, Hospital Universitario Río Hortega, Valladolid, Spain

**Keywords:** Mucopolysaccharidosis, Sly syndrome, Rare disease, MPS VII

## Abstract

**Background:**

Mucopolysaccharidosis type VII (Sly syndrome) is an ultra-rare neurometabolic disorder caused by inherited deficiency of the lysosomal enzyme β-glucuronidase. Precise data regarding its epidemiology are scarce, but birth prevalence is estimated to vary from 0.02 to 0.24 per 100,000 live births. The clinical course and disease progression are widely heterogeneous, but most patients have been reported to show signs such as skeletal deformities or cognitive delay. Additionally, detection criteria are not standardized, resulting in delayed diagnosis and treatment.

**Methods:**

We present a cohort of 9 patients with mucopolysaccharidosis VII diagnosed in the Iberian Peninsula, either in Spain or Portugal. The diagnostic approach, genetic studies, clinical features, evolution and treatment interventions were reviewed.

**Results:**

We found that skeletal deformities, hip dysplasia, hydrops fetalis, hepatosplenomegaly, hernias, coarse features, respiratory issues, and cognitive and growth delay were the most common features identified in the cohort. In general, patients with early diagnostic confirmation who received the appropriate treatment in a timely manner presented a more favorable clinical evolution.

**Conclusions:**

This case series report helps to improve understanding of this ultra-rare disease and allows to establish criteria for clinical suspicion or diagnosis, recommendations, and future directions for better management of patients with Sly syndrome.

## Background

Mucopolysaccharidosis type VII (MPS VII), also known as Sly syndrome, is a progressive neurometabolic disorder caused by a congenital deficiency of the lysosomal enzyme β-glucuronidase (*GUSB*). The disorder leads to progressive storage of the glycosaminoglycans (GAGs) dermatan sulfate, heparan sulfate, and chondroitin 4-,6-sulfate in the lysosomes of various tissues [1]. Since GAGs have many important cellular roles, their accumulation produces dysfunctional cells, generating a very heterogenous range of symptoms and phenotypes that affect many organs, including the central nervous system (CNS), resulting in multisystem damage. At a genetic level, inheritance is autosomal recessive, with most enzyme defects due to missense mutations. Moreover, the type and location of the mutations dictates the level of enzyme activity and, consequently, the severity of symptoms. MPS VII is an ultra-rare disease and precise epidemiological data are scarce, but birth prevalence is estimated to vary from 0.02 to 0.24 per 100,000 live births [2]. The first clinical case was recorded in 1973 by William S. Sly, who gave name to the syndrome, but few reports have been published since then. We herein present specific data regarding the Iberian population.

The clinical presentation and disease progression of MPS VII shows a wide spectrum, and affected patients may present from early, severe, multisystem manifestations to milder symptoms with later onset. Most patients with MPS VII have severe symptoms such as skeletal dysplasia, hepatosplenomegaly and cognitive impairment, which reduce patient quality of life and life expectancy [3]. Moreover, growth delay starting at around 2 years old has been described in these patients. Some MPS VII patients may present hydrops fetalis in utero or at birth, and most reported cases with this condition only survive for a few months. However, patients with milder manifestations usually present better outcomes, and some have even survived into the fifth decade of life [4].

As with other rare diseases, diagnosis of MPS VII may be difficult, and, in the absence of non-immune hydrops fetalis (NIHF), can go undiagnosed for years. In cases with clinical suspicion, the patient should be referred to a medical specialist for urinary GAG testing followed by β-glucuronidase activity testing. However, some countries are also implementing screening programs based on enzyme activity tests performed on blood cards or urine samples. One such targeted screening program—known as the FIND project—was launched in 2014 in Spain in collaboration with the Spanish MPS Association and with the endorsement of the Spanish Federation for Rare Diseases (FEDER) [5]. Genetic sequencing that searches for mutations in the *GUSB* gene can be used to confirm the diagnosis, enabling genetic counseling to be offered to the family [4, 6].

In addition to supportive treatment, the currently available therapies for MPS VII are enzyme replacement therapy (ERT) [7] or bone marrow transplant. ERT is administered using a recently developed recombinant form of human *GUSB* (vestronidase alfa) that has been used successfully to reduce urinary GAGs and improve organomegaly [8]. Pharmacokinetic studies have supported the recommended 4 mg/kg QOW (once every other week) dosing regimen of vestronidase alfa both for pediatric and adult MPS VII patients [9]. Since ERT has no effects on neurological signs (it does not cross the blood–brain barrier), hematopoietic stem cell transplantation (HSCT) with bone marrow or umbilical cord blood stem cells has been performed, as in other lysosomal diseases, showing diverse outcomes. While early HSCT has slowed or prevented neurological deterioration in some patients, others have died because of infection or development of graft-versus-host disease [10].

The aim of this study was to expand current information regarding the diagnosis, clinical course, and treatment of MPS VII syndrome. We have provided data from 9 patients belonging to the Iberian cohort (Spain and Portugal), and discussed their common symptoms and interventions, which may help to better understand this ultra-rare disease and allow criteria and recommendations to be established for better patient management.

## Clinical case series

A summary of the most important clinical characteristics of the patients is shown in Table 1.

**Table 1:** Epidemiological and clinical features of MPS VII Iberian cohort

Patient	Age at diagnosis/sex	Clinical manifestations	MPS VII treatment
1	8 years/M	Cognitive delay, coarse face, quadriplegia, chest deviations, thoracic kyphosis	None
2	12 months/F	Clubfoot, hip dysplasia, scoliosis, mild aortic thickening, corneal clouding, respiratory issues	Bone marrow and umbilical cord transplants
3	2 years/M	Coarse face, dysostosis, splenomegaly, macrocephaly, psychomotor delay, hip dysplasia, hernias	Vestronidase
4	14 months/M	Hydrops fetalis, hypospadias, skeletal deformities, scoliosis, hepatic hemangiomas	Vestronidase
5	18 months/M	Hydrops fetalis, breathing issues, cognitive and growth delay, severe scoliosis, mild valvular deficiency	Vestronidase
6	2 years/M	Severe trunk issues, lower limb hyperreflexia, hepatomegaly, hernias, cognitive delay	Vestronidase
7	26 months/F	Hydrops fetalis, hip dysplasia, cognitive delay, ENT issues, spinal deformities, osteoporosis	Vestronidase
8	5 months/M	Dysmorphic signs, hepatomegaly, hernias, developmental delay, heart hypertrophy	None
9	3 months/M	Hepatomegaly, psychomotor delay, epilepsy with focal seizures	Vestronidase

F: Female; M: Male; ENT: ear, nose, throat; MPS: Mucopolysaccharidosis

### Patient 1

This patient was previously described in Montaño et al. 2016 [3]. Patient 1 was a 3-year-old male with psychomotor delay. At the age of 6, he was enrolled in a special school due to moderate cognitive delay. When he was 8 years old, he showed coarse facial features, pectus excavatum and bone dysplasia. Sly syndrome was diagnosed after detecting elevated GAG levels (Table 2). When he was 10 years old, he underwent bilateral keratoplasty surgery with ocular anesthesia. At age 12, he developed flaccid quadriplegia with dislocation of C1–C2 after a somersault with sudden flexion of the head in gymnastics. Emergency surgery with suboccipital craniotomy was performed with C1–C4 laminectomy, fixing C5–C6 with a rod. Some weeks later, he was immobilized as a result of spastic quadriplegia, particularly on the right side, and chest protrusion was evident. Clinical exam showed macrocephaly, coarse face with prominent jaw, saddle nose, inverted nasal wings, bushy eyebrows, exophthalmos, low ears, and short neck. Other signs such as abnormal dentition, protruding sternum and hepatosplenomegaly were also observed. X-ray revealed spondyloepiphyseal dysplasia, joint stiffness, deformation of vertebrae in parrot beak, thickened ribs and clavicula, and hip/odontoid dysplasia. At 14 years old, after two years of physiotherapy, he was able to walk again with residual slight right hemiparesis, but later hamstring retraction required surgery. The patient had a dental abscess at age 18 removed without anesthesia. One year later, he showed severe thoracic kyphosis and major retractions. His parents did not want to participate in a vestronidase clinical trial due to the difficulties entailed in taking him to another country. He is now 36 years old, wheelchair-bound, and his cognitive level has decreased to an intelligence quotient (IQ) of 35.

**Table 2:** Summary of molecular data for the MPS VII Iberian cohort

Patient	Age at diagnosis	*GUSB* mutation	Amino acid Change	Exon	β-glucuronidase activity at diagnosis from leukocytes or fibroblasts nmol/h/mg protein (ref. range)	GAG levels at diagnosis mg/mmol/creatinine (ref. range)
1	8 years	NA	–	–	NA	16,7 (2,2–5,7)
2	12 months	c.50G > C c.1325C > T	p.W17S p.A442V	1 8	3,17 (268–1323)	50,80 (0,65–12)
3	2 years	c.526C > T Homozygous	p.L176F	3	0,6 (NA)	41 (NA)
4	14 months	c.1491 T > G c.1747G > A	p.F498V p.R582L	10 11	2 (147–1148)	NA
5	18 months	c.530C > T Homozygous	p.T177I	3	1 (NA)	NA
6	2 years	NA	–	–	26.04 (268–1323)	Normal^a^
7	26 months	NA	–	–	18 (147–1148)	NA
8	5 months	c.1387 T > G Homozygous	p.L463V	8	2.4 (96–233)	NA
9	3 months	c.1486G > T c.1760G > C	p.A496S p.V584A	10 11	1.7 (96–233)	115 (6–22)

GAG: Glycosaminoglycans; NA: Not available

^a^No data was obtained from the patient’s referral center

### Patient 2

Patient 2 is a female born premature at 34 weeks of pregnancy, with right congenital talipes equinovarus foot (clubfoot) with good progress using the Ponseti method. Patient’s characteristics were previously described in Montaño et al. [3]. Esotropia was monitored by the ophthalmology department. She used cranial orthosis for plagiocephaly at 10 months of age. At age 7 months, she presented dislocation of the right hip due to right femoral coxodysplasia. She remained in hospital with a pelvic plaster due to her hip dysplasia until she was 12 months old. At 1 year old, the orthopedist consulted the neurology department due to an X-ray of the spine that showed vertebrae with a parrot beak appearance; mucopolysaccharidosis was suspected. The metabolic study showed decreased β-glucuronidase activity and accumulation of GAGs. At 15 months old, genetic testing showed a heterozygous mutation in the *GUSB* gene (Table 2). The patient did not have any hernias but showed mild scoliosis. Her cognitive level and height were normal until 1 year old. She attended physiotherapy and walked at 22 months old. She received her first allogenic donor bone marrow transplant when she was 26 months old. Four months after the transplant, an increase in enzyme activity was observed (555 nmol/h/mg protein [268–1323]), but GAG levels remained high (19.03 mg/mmol/creatinine [0.26–7.07]). At 6 and 9 months, she presented two episodes of graft-versus-host disease. Infusion of donor lymphocytes did not resolve the problem, and 6–9 months after the transplant, the host graft was rejected. Around a year later, at age 3 years and 11 months, she underwent successful umbilical cord blood transplant, retaining high levels of beta-glucuronidase activity, low GAG levels and full donor chimerism, which has remained until now. At the age of 7, she showed no skeletal retractions but presented mild aortic thickening and slight corneal clouding. She was reactive for Epstein-Barr disease and has received gamma globulin treatment every 2 weeks since she was 9 years old due to reactivation of Epstein Barr virus and IgG deficiency because she has no memory B lymphocytes as a result of the transplant [11]. At 10 years old, her scoliosis worsened, and thoracolumbar nocturnal orthosis was applied. She also underwent surgery due to an umbilical hernia. She is currently 12 years old, with a normal cognitive level for her age. She has followed physical therapy (both motor and respiratory) and occupational therapy in our hospital. The pain in her hip and left knee made it difficult to walk; consequently, she had surgery of the left hip subluxation at 12 years of age.

### Patient 3

This was a male patient born at term after a normal pregnancy. At birth, he showed coarse face, short neck, and macroglossia in addition to other symptoms, including severe dysostosis and splenomegaly. He was extensively investigated. He was referred to Metabolic Investigation at 2 years old with mobility issues, macrocephaly, mild psychomotor retardation and hernias, and his growth rate had plateaued. MPS was suspected; levels of β-glucuronidase activity were low (0.6 nmol/h/mg protein) and genetic testing found a homozygous mutation in exon 3 of the *GUSB* gene, confirming Sly syndrome when he was 26 months old. Patient symptoms worsened with age. He suffered repeated airway infections for which he had undergone two otorhinolaryngological surgeries at the ages of 2 and 4 years old. He also underwent surgery for a hernia when he was 3 years old. An echocardiogram at 4 years old showed slight distal thickening of the mitral valve leaflets (without stenosis or regurgitation). He also presented skeletal abnormalities, with dysmorphia in most of the vertebral bodies and severe hip dysplasia. Vestronidase treatment was initiated when he was 4.5 years old. Since then, his clinical condition has improved; he in integrated at school with moderate cognitive impairment; his growth has stabilized, splenomegaly has reduced, dental abnormalities have decreased, and he has no corneal clouding. The patient is currently 8 years old, and still has severe hip dysplasia with severe walking difficulties.

### Patient 4

**Fig. 1:**
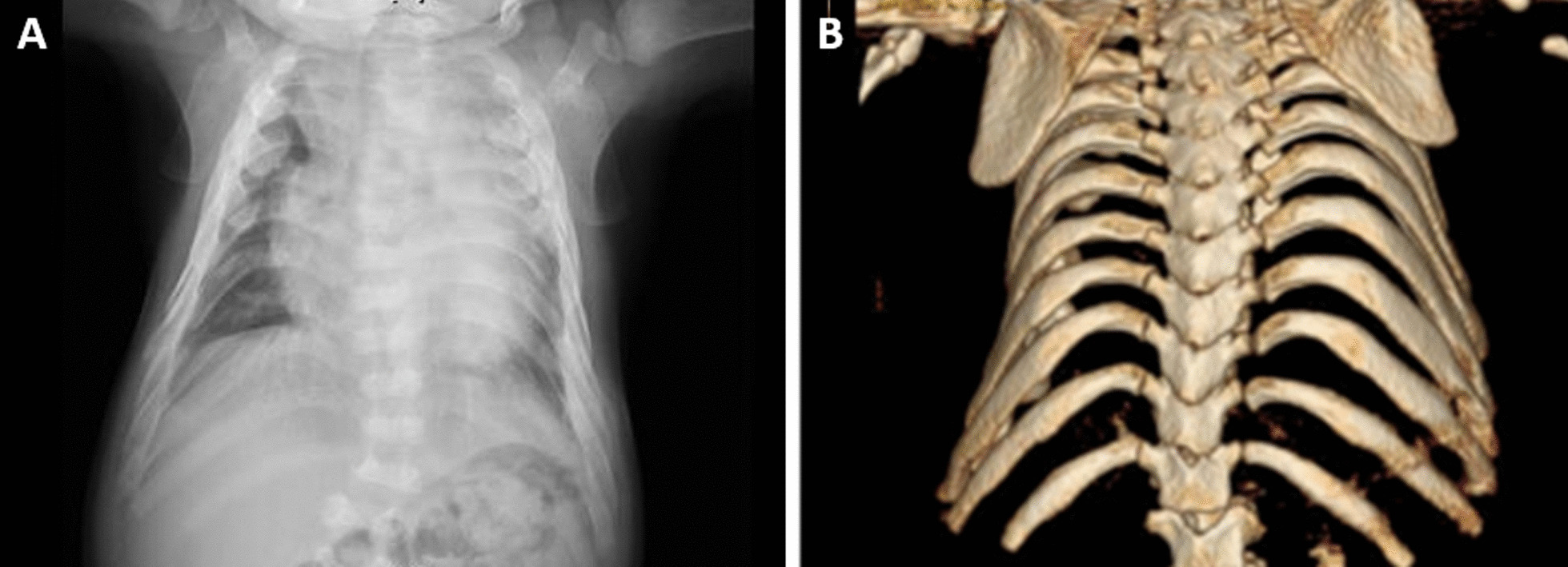
X-ray and MRI images of a MPS VII patient. **a** Marked cardiomegaly, and thoracic deformity causing a significant loss of volume of the left lung parenchyma. **b** Small vertebral bodies, with hook-like morphology

This was a severe case of a male neonate born with hydrops fetalis, hypospadias, portosystemic shunt and a 47 XYY karyotype detected prenatally. At birth, he was also diagnosed with neonatal lung disease of unknown origin, neonatal persistent pulmonary hypertension, non-compaction heart disease, portosystemic fistula, and sensory hearing loss. At 7 months, he had a bronchiolitis infection. He suffered chronic restrictive respiratory failure secondary to thoracic malformation, requiring oxygen therapy during the day in periods of infection and continuous non-invasive ventilation, and using bilevel positive airway pressure machine (BIPAP) when sleeping. The radiologist observed a deformity of the rib cage and the coarse shape of the ribs when he was 12 months old (Fig. 1). He also presented cardiomegaly, dorsolumbar scoliosis, and long bones with widened metaphyses. When he was 1 year old, he was referred to a specialist from his local hospital and MPS VII was suspected after analyzing his clinical data. GAGs and enzyme activity were measured, confirming the diagnosis of Sly syndrome at 14 months. He started ERT with vestronidase at 17 months. After 15 months of treatment (31 doses of the enzyme therapy), there has been a substantial improvement in all facets of neurodevelopment. His motor skills are good, and he has some single words of verbal communication and adequate understanding of non-complex sentences. There has been a significant decrease in respiratory complications derived from viral infections and during sleep periods, allowing BIPAP to be withdrawn at night. He does not require supplemental medication for lung function. He has recently undergone surgery for hypospadias and bilateral tubal drainage, with no peri- or postoperative incidents.

### Patient 5

Male patient born with hydrops fetalis, hepatosplenomegaly with thrombocytopenia and abnormal fat pad. He was diagnosed at 18 months with Sly syndrome after finding low β-glucuronidase activity levels. A homozygous mutation in *GUSB* gene confirmed the diagnosis (Table 2). As a child, he presented important breathing issues leading to chronic respiratory insufficiency under non-invasive ventilation. He showed progressive skeletal deformities of the thorax, spine, hip, and knee joints with short stature. His cognitive development was delayed as well. During his childhood he also suffered epilepsy attacks that were controlled with levetiracetam. At 14 years old he presented severe scoliosis with compressions. Additionally, he showed some valvular deficiency but no cardiac insufficiency. Treatment with vestronidase was started in the USA at 10 years old and continued in Portugal (2015). The organomegalies improved and the osteoarticular involvement stabilized. He has severe cognitive impairment. He emigrated to Germany in 2019 and treatment was stopped in 2020 at the mother’s request.

### Patient 6

This was a male patient with trunk shortness, lower limb hyperreflexia and hepatomegaly. Although the first GAG measurement showed normal levels, there was a strong clinical suspicion of metabolic disease and glucuronidase activity assays confirmed MPS VII when he was 24 months old. Stem cell transplant was not considered at this time for this patient. He participated in the Phase I vestronidase clinical trial. At age 5, he suffered cervical cord compression during a surgery because of spine instability, with mobility sequelae and respiratory support. He continued receiving vestronidase and is now able to breathe without any support and has recovered movement in the upper limbs. His disease has stabilized except for the CNS involvement, as he has some developmental delay. He continues with treatment with good tolerance, with no treatment-related adverse events.

### Patient 7

Female born with hydrops fetalis after a normal pregnancy. MPS VII was suspected as she presented the typical phenotype, confirmed with β-glucuronidase activity assays when she was 26 months old. At age 4, she presented hip dysplasia and cognitive delay as well as many otorhinolaryngologic problems. Neither hepatomegaly nor splenomegaly was observed, and she had no hernias. Several spine anomalies with progressive scoliosis were detected, in particular retrolisthesis C7-T1. Anterior–posterior surgery was performed to fix cervical-thoracic scoliosis (C2-T4). She has been receiving vestronidase treatment for around 100 weeks now, which is well tolerated, except for a non-itchy fixed drug eruption that appears in the arm-elbow joint while she receives the treatment, although it only lasts around 30 min before disappearing. Today (age 8 years), the patient has mild cognitive delay, and is a very active girl (424 m in the last 6-min walk test), despite hip dysplasia and osteoporosis. She needs to undergo further surgery to replace the surgical pins in her spine. The transplant option has not been considered by the parents yet.

### Patient 8

Male patient born in 1992 after full-term pregnancy of non-consanguineous parents. His birth weight, height, and head circumference were within normal limits. He presented dysmorphic signs, hepatomegaly and inguinal bilateral and umbilical hernias, suggestive of a metabolic storage disease. Levels of β-glucuronidase activity in leukocytes and fibroblasts (2.4 nmol/h/mg protein [96–233] and 1.3 [19–111], respectively) measured at 5 months old were compatible with a diagnosis of MPS VII. He was hospitalized multiple times because of upper and lower respiratory tract infections, often with a bronchospastic component. At 18 months, obstructive apneas were confirmed by polysomnographic study. At 6 years old, the patient presented significant psychomotor developmental delay with hyperactivity, impulsivity and aggressiveness, treated with risperidone. He also presented visual and auditory deficit, and heart hypertrophy with cardiac valve dysplasia. When he was 14 years old, he was hospitalized due to polyarthralgia of the large joints and persistent fever. Septic arthritis of the left hip developed, and he underwent surgery.

Trans-esophageal echocardiogram showed slight pericardial effusion and minor mitral valve insufficiency. *Staphylococcus aureus* septicemia, with anemia, diffuse interstitial pneumonia, respiratory and renal failure led to death, in spite of intensive care.

Recent study of the *GUSB* gene in the patient’s fibroblasts revealed a not yet described pathogenic variant in homozygosity in exon 8 (Table 2).

### **Patient 9 (Fig. ****2****)**

**Fig. 2:**
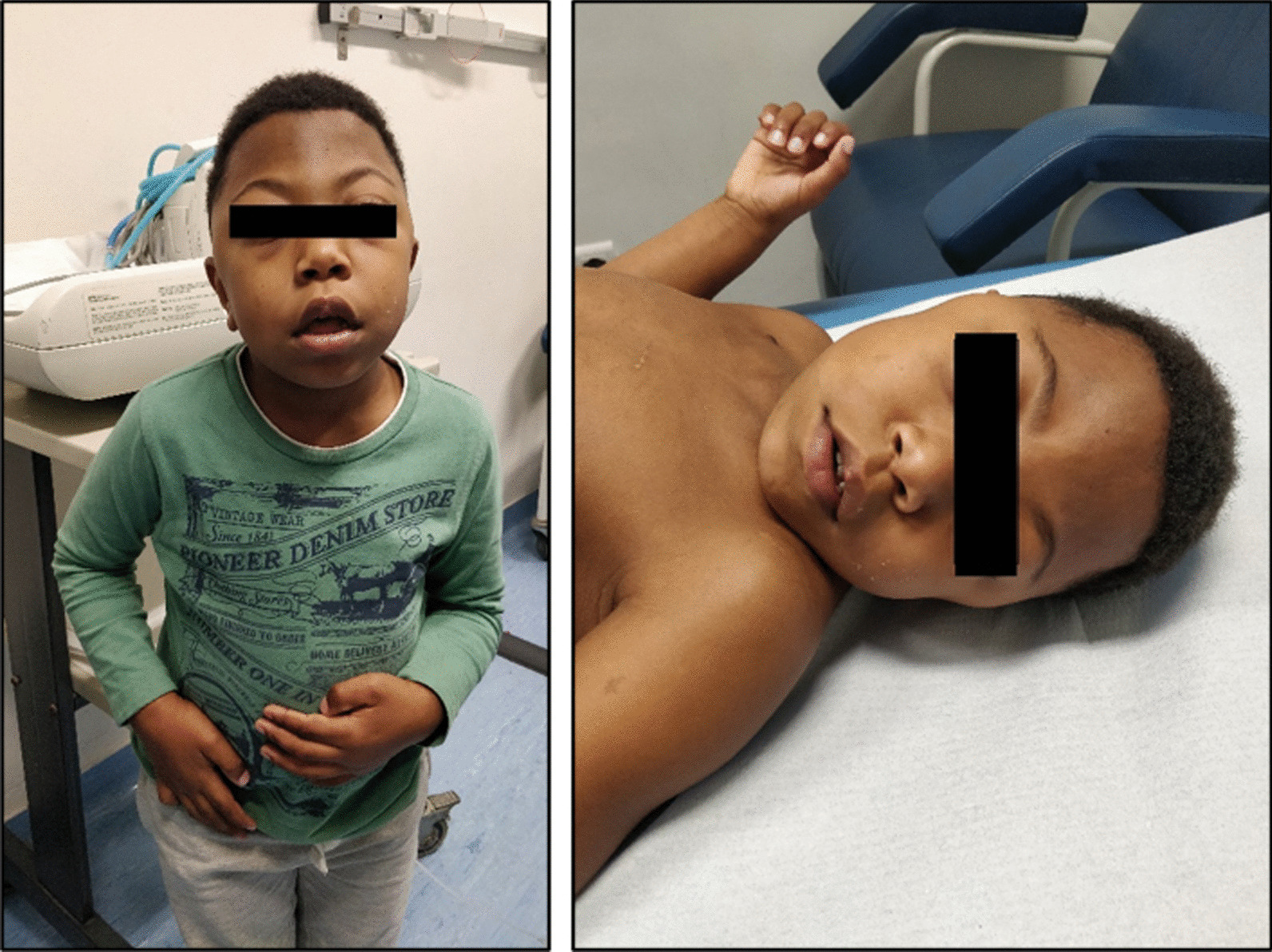
Dysmorphic appearance and facial features of a MPS VII patient (patient 9)

Five-year-old boy, second son of a non-consanguineous couple, born after an uneventful pregnancy. On the first day of life, he had hypoglycemia and seizures, which required phenobarbital. Electroencephalogram was normal and brain MRI showed posterior cortico-subcortical lesions. Hepatosplenomegaly with cholestatic hepatitis and mild tricuspid valvular insufficiency with a slight hypertrophic interventricular septum (5.3 mm) were also noted in the neonatal period. Extensive etiological investigation identified high urinary GAGs and low levels of β-glucuronidase measured in lymphocytes suggestive of MPS VII, confirmed by molecular test (Table 2). At 4 months old, dysmorphic features were noted, characterized by a prominent forehead, gingival hypertrophy, umbilical hernia and persistent hepatomegaly. Overall developmental delay became evident at 9 months old, so the patient began speech and occupational therapy. During follow-up, he had frequent respiratory infections with bronchospasm, dysmorphic features became more pronounced, and he had focal seizures controlled by carbamazepine treatment. ERT with vestronidase was started at 15 months old, with no adverse events reported to date. A favorable evolution has been reported in terms of neurocognitive development and improved respiratory status. Currently, he only has sporadic seizures (twice per year), has mild tricuspid and mitral valvular insufficiency, no corneal opacities, and no hepatomegaly or splenomegaly.

## Discussion

We present herein a series of 9 clinical cases of MPS VII in the Iberian Peninsula, focusing on clinical symptoms, genetics, diagnostic algorithm and treatments used. To our knowledge, this is one of the largest case series studies in a localized cohort presented for this disease.

As with other MPS, Sly syndrome is a very heterogenous disorder with variable clinical presentations, although in most cases it is a severe and progressive disease [12]. The most common signs found in this cohort were skeletal deformities, hip dysplasia, hydrops fetalis, hepatosplenomegaly, hernias, coarse face features, respiratory issues, cognitive delay and failure to thrive (Table 1). This is consistent with previously reported data worldwide [1, 4].

Morrison et al. reported in their survey how the symptoms that are most likely to raise suspicion of MPS VII, in the absence of NIHF, typically do not appear until age 3 or older [4]. In our cohort, three of the patients were confirmed to have Sly syndrome before the age of 1. Moreover, most of the MPS VII patients in this study were diagnosed before they were 3 years old (8 out of 9), and half of them did not present NIHF. This could be due to slightly enhanced awareness of the disease among healthcare professionals, thanks to screening programs such as the FIND project. These programs are helping to identify the earliest symptoms, thus prompting swift referral to the appropriate specialist so that confirmatory tests can be performed as soon as possible. As with many other diseases, this is very important, since rapid diagnosis will allow early initiation of therapy, potentially halting disease progression and minimizing its consequences. Even today, treatment does not start early enough in many cases, and patients often require surgery, with the subsequent risks of anesthesia [13]. Furthermore, according to the literature, the MPS VII phenotype is harder to recognize in older patients than in young ones [3].

A few recommendations for a more solid diagnostic approach may arise out of this case series. In neonates and young patients (< 2 years old), the neonatologist and pediatrician should follow the appropriate MPS screening method described by each center when metabolic disease is suspected [14]. Additionally, in the presence of NIHF, specific genetic testing is recommended. In older patients (> 2 years of age), short stature and the presence of skeletal dysplasia, especially with hepato- or splenomegaly, are manifestations that should lead physicians to perform extra tests to determine if the patient has MPS; cognitive delay, especially when combined with other manifestations, should also be considered. Skeletal abnormalities, which are usually associated with other lysosomal diseases, also appear to be an obvious sign of MPS VII, and result in loss of limb mobility. In older patients, cardiac signs should be also considered. Therefore, early detection of clinical manifestations together with genetic panels available at qualified centers likely represent the future, although simpler and quicker tests like measuring GAG levels or enzymatic activity may also be performed.

Most interventions carried out in the patients of this cohort were surgeries aimed at treating the signs derived from the disorder, mainly skeletal deformities. Only two patients underwent transplantation; despite the initial improvement, however, retaining high enzyme activity levels and low GAG levels, some symptoms worsened later.

With respect to ERT with vestronidase alfa, the six patients who received the treatment have shown significant clinical improvement with good tolerability of the therapy. Reports have shown reductions in hepatosplenomegaly, dental abnormalities and corneal clouding. Moreover, patients under vestronidase alfa treatment had also substantially improved their breathing issues and their growth rate had stabilized. Although vestronidase alfa does not cross the blood brain barrier, it was interesting to note that most of the treated patients had a favorable neurocognitive evolution. Since MPS VII is a severe disease with rapid progression, the clinical improvement observed after vestronidase alfa treatment is remarkable. Furthermore, patients, their families and caregivers have improved their quality of life as a result of the high effectiveness reported for vestronidase alfa treatment.

The high-resolution crystal structure of the human β-glucuronidase monomer revealed three distinct domains: Jelly roll barrel (residues 22–223) that include the lysosomal targeting motif (residues 179–201), an immunoglobulin region constant domain (residues 224–342), and a TIM barrel domain (residues 342–632) [15]. We obtained genetic data from six of the nine reported patients with MPS VII (Table 2). While some of them carried mutations in the *GUSB* gene that have been previously described (patient 2 and patient 3), other pathogenic variants were reported in this cohort for the first time, all of them missense mutations. Interestingly, in a recently published case series study of a Brazilian cohort, the homozygous mutation c.526C > T (p.Leu176Phe) found in patient 3 was also present in 12 of the 13 patients analyzed [16]. Among the new mutations observed, it is remarkable that patients 4 and 9 present changes in nearby regions belonging to exons 10 and 11, associated in all of them with a severe symptomatology. Intriguingly, those mutations found in exon 10 correspond to residues belonging to the TIM barrel domain that are either conserved or homologous between species [15], suggesting that these regions might be important for the functionality of β-glucuronidase.

Increasing awareness among specialists who treat MPS VII patients is one of the future pathways to follow. Most patients with Sly syndrome are diagnosed by the neonatologist or metabolic specialist. However, hip dysplasia, for example, was usually an evident sign of MPS VII among this Iberian cohort of patients. Thus, orthopedic physicians as well as orthopedic surgeons treating adult patients should be aware of the possibility of MPS VII, given that all these patients have metabolic skeletal dysplasia. Likewise, other specialists such as endocrinologists and cardiologists should receive training on MPS VII. Regardless of the specialist involved in the diagnosis, the patient should be managed by a multidisciplinary team who are sufficiently educated in MPS VII. This should include rehabilitation specialists, speech therapists, physical therapists, respiratory physicians, cardiologists, neurosurgeons, psychologists, ophthalmologists, ENT specialists, occupational therapists, orthopedic surgeons and anesthesiologists.

This case series report gives some insight into the common signs and clinical evolution of patients with MPS VII, as well as their relationship with the diagnostic approach. Further research studies focusing on MPS VII are needed to improve management and outcomes for these patients. For example, studying the association between genotype and phenotype, specifically to associate mutations with the severity of the disease, could be used like in other MPS diseases as a criterion to determine which patients would be candidates for bone marrow transplantation.

## Conclusion

Despite the heterogeneous course of MPS VII, there are common signs such as skeletal deformities or cognitive delay that should always raise suspicion of this disorder, allowing for appropriate diagnostic tools to be implemented as soon as possible. Early detection, management by a multidisciplinary team and further research to standardize diagnostic algorithms are key determinants to improve patient outcomes and clinical evolution.

## Data Availability

The datasets generated and/or analyzed during the current study are not publicly available due to their containing information that could compromise the privacy of participants but are available from the corresponding author on reasonable request.

## Abbreviations

CNS: Central nervous system
ERT: Enzyme replacement therapy
GAG: Glycosaminoglycans
GUSB: Lysosomal enzyme β-glucuronidase
MPS: Mucopolysaccharidosis
NIHF: Non-immune hydrops fetalis
